# Supplementary material for: Pannexin 1 Transgenic Mice: Human Diseases and Sleep-Wake Function Revision
Source: Int J Mol Sci. 2021 May 17;22(10):5269. doi: 10.3390/ijms22105269 (PMC8155943; doi:10.3390/ijms22105269)
Supplement: Supplementary file 1 [file ijms-22-05269-s001.zip › ijms-1196043-supplementary.pdf]

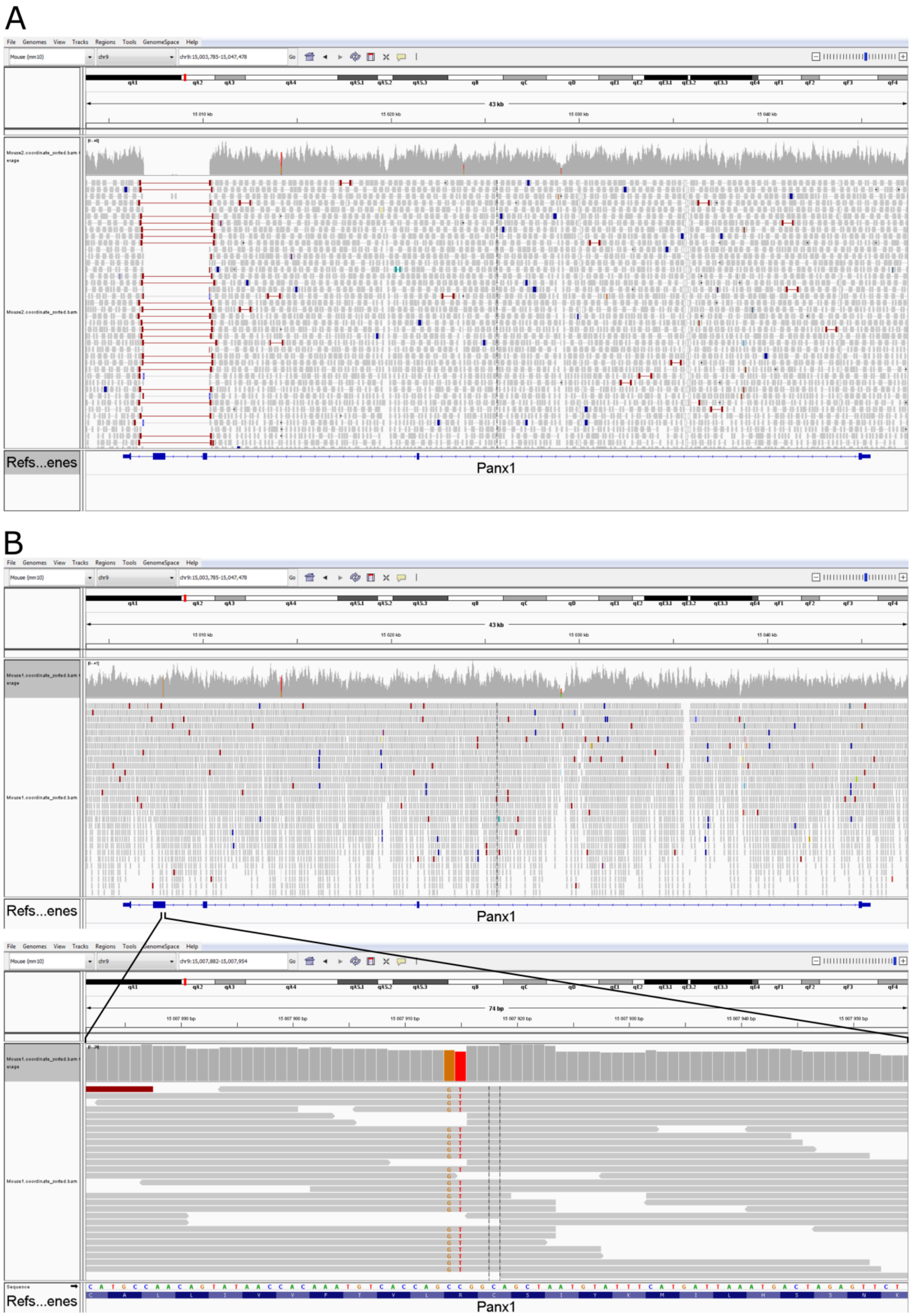

Figure S1: The results of whole-genome sequencing for mice from Knockout (A) and Substitution (B) groups.
